# The “Writing Spiral”: A Practical Tool for Teaching Undergraduates to Write Publication-Quality Manuscripts

**DOI:** 10.3389/fpsyg.2019.00915

**Published:** 2019-04-24

**Authors:** Traci A. Giuliano

**Affiliations:** Department of Psychology, Southwestern University, Georgetown, TX, United States

**Keywords:** undergraduate research, undergraduate publishing, teaching writing, undergraduate writing, writing skills

## Introduction

One of the perceived barriers to publishing with undergraduates (especially with undergraduates as first author; see Giuliano, 2019) is the concern that students lack the requisite writing skills to make a significant contribution. For example, several authors in a recent special issue devoted to publishing with undergraduates (see “Engaging Undergraduates in Publishable Research: Best Practices,” *Frontiers in Psychology*) discuss this challenge (e.g., McKelvie and Standing, 2018; Detweiler-Bedell and Detweiler-Bedell, 2019; Reavis and Thomas, 2019; Scisco et al., 2019). Unfortunately, despite the numerous individual articles describing tips for teaching writing in psychology (in journals such as *Teaching of Psychology*), as well as step-by-step books that teach students to improve their writing, there is no widely-accepted comprehensive resource to guide faculty in teaching psychological writing to students (see Ishak and Salter, 2017, for a review). The purpose of this paper is to address this gap, with a focus on teaching undergraduates to write publication-quality manuscripts. Although there are many important factors in teaching writing, one tool that I have developed—which students call “the writing spiral”—has been successfully used to guide numerous undergraduates through the publication process^1^. After describing the purpose and benefits of the writing spiral, I provide a detailed description of its contents in the hopes that interested readers might successfully incorporate some of this material into their own writing instruction^2^.

## Purpose and Benefits of the Writing Spiral

As an undergraduate, I always appreciated detailed handouts and clear expectations for assignments, so it is not surprising that as a professor I would become well-known for my prolific handouts. A handout typically is “born” when I realize that my frustration that students are not producing higher quality work on an assignment could be ameliorated by giving them more clear expectations and examples. Indeed, each time I have produced a new handout, I have been rewarded with improved student work. Early in my career, many of these handouts originated from students' lack of skills in grammar, writing mechanics, scientific tone, and APA style—especially in my research methods course. Although some discourage teaching such mechanics in discipline-specific writing (e.g., arguing that it is time consuming, distracts from content, and that students should be willing, and able to achieve competence in these areas on their own; Willingham, 1990), my experience has been consistent with research showing that direct training in grammar and APA style significantly improves students' skills and confidence (see, for example, Goddard, 2003, who found significant positive changes in students' attitude toward writing, confidence in writing empirical papers, and skills in grammar and APA style following a writing course for psychology majors).

Over time, the handouts became longer and more numerous, and the writing spiral was created in response to my research methods students' request that I collate the handouts (which were posted online as separate documents) into a single printed resource that they could carry with them. The benefits of the writing spiral are many: First, in addition to what students learn from the contents, they are much more likely to actually use the handouts in this form (they report that they always have the spiral open while they are researching and writing papers). In addition, students also learn a general consistency in writing style and convention specific to me that is helpful if they later take my capstone research course (a junior/senior level course in which 5–6 students collaborate with faculty for two consecutive semesters)^3^. Finally, the spiral is used by students in their capstone courses, in other psychology courses, as well as when pursuing post-graduate degrees in psychology or related fields (e.g., social work, counseling, nursing, medical school).

## Contents of the Writing Spiral

The writing spiral is officially titled “Dr. G's Guide to Writing, Grammar, and APA Style” and consists of a collection of handouts printed on different-colored paper and bound into a spiral with a clear cover over the index (The writing spiral can be downloaded at https://drive.google.com/file/d/1dYWhky4FaJ9jepBAj29JXUsbQbLY2ytc/view?usp=sharing). It contains 10 handouts as follows:

1. **Southwestern University Guide to Writing in Psychology**. I wrote this brief guide as a resource for our campus writing center, which asks each department to develop a disciplinary writing guide. This guide describes tips for writing common types of assignments in psychology (e.g., a literature review, a research report, a journal critique), a description of rules about evidence and citation, and a “*Do*'s” and “*Don't*s” list (with examples) of discipline-specific writing conventions and formatting (e.g., APA style).
2. **Writing Competently Grammar Handout**. This handout consists of a section on grammar from the “Writing Competently” chapter in the *Psychology Student Writer's Manual* (Scott et al., 2002). In particular, I like its description of parallelism, comma splices, fragments, vague pronoun references, and other word-choice errors (e.g., *since* vs. *because, while* vs. *although/whereas*) that are common (but rarely understood) mistakes made by my students^4^.
3. **Intro to APA Style/APA Template**. Because research shows that intensive instruction in APA style leads to improved skills and is a precursor to better scientific writing (e.g., Goddard, 2003; Fallahi et al., 2006; Luttrell et al., 2010), there are three handouts on APA style in the spiral. Unfortunately, I am unable to locate the source for this first handout, which I encountered during graduate school at UCLA almost three decades ago. It cleverly describes many APA rules and conventions, all in a format that is itself in an APA-style paper, thus providing a good, gentle introduction to APA style for students.
4. **Dr. G's Guide to APA Citation**. I wrote this handout (which has evolved significantly over the years) to help students understand some of finer points of APA citation, including what secondary citations are and why they should be avoided, how to write in a way that minimizes tedious citation (i.e., the same citation in parentheses after several sentences in a row), and the difference between “word” and “idea” plagiarism, which I have found improves students' understanding of plagiarism in general.
5. **Dr. G's Step-by-Step Guide to Writing an APA Paper**. This handout teaches students that APA style is much more than idiosyncratic formatting rules, and that it helps with paper organization (Goddard, 2003), structure (i.e., the “hourglass” shape recommended by Bem, 2003), and content (i.e., the “recipe” or formula for each section is described and then supplemented with examples from my own published research). Although I still require students to consult the APA manual, this handout is one of the most frequently used in the spiral, and provides a starting point for writing each section of a paper.
6. **Dr. G's Manuscript Comment Codes**. I developed this one-page handout—which contains the “codes” or abbreviations that I write on student papers while grading (such as “PC” to indicate a parallel construction error, “Ch” for choppiness, or “Cas” for casual/informal language), as well as examples of each—both to save myself the extra writing of explaining the same comments over and over and to help us develop a consistent language (i.e., a “shared understanding” of feedback; Glover and Brown, 2006) for discussing writing issues in my courses (see also Beins et al., 2010).
7. **Dr. G's Turds in the Punchbowl**. Borrowing an idea from an English department colleague who famously says that bad writing, much like a “turd floating in a punchbowl,” tends to “spoil the party,” I've compiled a list of words and phrases frequently used by my students that are either grammatically incorrect or awkward (e.g., “In congruence with the hypothesis,” “the researcher *states/goes on to say* that…”), or that violate scientific convention or tone (e.g., “the results *prove* that…,” “the results were *insignificant*”). Interestingly, students who introduce new terms to the list in their own papers are surprisingly honored to make the next year's version of the handout.
8. **Dr. G's Transitions Cheatsheet**. Smooth flow is incredibly important to a paper's readability, but I find that students have rarely been taught to use transitions in their writing. Thus, in this one-page handout, I briefly explain the difference between transition *sentences* (which are used to logically link the ideas in one paragraph to the ideas at the beginning of a subsequent paragraph) and transition *words and phrases* (which are used between sentences to prevent “choppiness” and improve flow). Several examples of transition words and phrases (e.g., “As such,” ‘That is,” “In a similar vein,” “Specifically”) are grouped together in this handout by common meaning for students to use as they write. Although students sometimes try to use transition phrases interchangeably despite differences in meaning, with feedback and practice even the weakest writers begin to write much smoother, easier-to-read papers.
9. **Dr. G's Discussion Phrases Cheatsheet**. Because writing empirical papers in psychology follows a pretty specific formula or “recipe,” and because many beginning writers do not have enough expertise to know the “ingredients,” I provide students with examples of common phrases that good writers use in specific situations, an approach advocated by Graff and Birkenstein (2014) in their best-selling book, *They Say, I Say: The Moves That Matter in Academic Writing* (I also use their approach to teach writing in my first year seminar course; see Giuliano, 2014). These template phrases include choices for several parts of the discussion section, including linking findings to previous research (e.g., “This pattern of results is consistent with previous literature showing that…”), introducing limitations (e.g., “Although the present results offer clear support for…, it is appropriate to recognize several potential limitations”), discussing practical implications (e.g., “Despite these limitations, our results suggest several practical implications”), and making suggestions for future research (“In terms of future research, it would be useful to extend the current findings by examining…”).
10. **Dr. G's Sample Student Manuscript in APA Style**. The final handout contains a recently-published article (Matthews et al., 2018) that was first-authored by a past student based on a research methods class project. The paper was created in Word format and is printed in double-spaced, APA-style manuscript form (rather than in the single-spaced, two-column format as it appears in the journal) so that students not only have an excellent content model to follow, but they can easily follow the formatting example for an APA-style manuscript (see also Ware et al., 2002). I have found that using a publication from a previous student shows current research methods students that writing publication-quality manuscripts (and subsequently publishing papers) is possible.

## Conclusion

Although some might think my approach is too heavy-handed (certainly reasonable people can disagree about the best strategy for teaching writing), my experience has shown that the use of the writing spiral dramatically increases students' writing skills and has led to many co-authored (*n* = 30 papers involving a total of 73 undergraduates)—and especially first-authored (*n* = 25)—student publications (see Giuliano, 2019). Consistent with Fallahi et al. (2006), who concluded that student-friendly models of teaching basic writing skills are well-worth the time and effort, students report that the writing spiral is a convenient tool that helps them become stronger writers overall (in both psychology and non-psychology courses, and even in graduate school and beyond). On end-of-semester course evaluations, the writing spiral has received extremely positive ratings thus far (i.e., the average rating for both “usefulness” and “recommend keeping” is 5.0 out of 5.0 for the two semesters that I've used the spiral; *n* = 19). Even years after students graduate, they email me to tell me that they are still using the spiral, and that they are sharing it with their graduate school colleagues who were not as fortunate to receive strong writing training during their undergraduate careers. Ultimately, the writing spiral helps counteract two common concerns that faculty have about teaching writing-intensive courses, namely the increased workload and negative reactions from students about writing (Boice, 1990). In short, the writing spiral decreases my workload while improving student writing in a way that is helpful and less unpleasant to them.

## Author Contributions

The author confirms being the sole contributor of this work and has approved it for publication.

### Conflict of Interest Statement

The author declares that the research was conducted in the absence of any commercial or financial relationships that could be construed as a potential conflict of interest.

## Footnotes

^1^I include my track record of publications with students later in this paper as suggestive evidence of the spiral's effectiveness; however, because I've used these handouts for much of my career, I didn't have the opportunity to collect empirical data (e.g., before and after the use of these handouts) to support my claims, which are primarily based on my own experiences guiding students to publication (as well as their self-reports). Future research (e.g., conducted by faculty who adopt the spiral in their work with students) would be helpful to verify the empirical effectiveness of the writing spiral.

^2^Please include a footer on any handouts based on these materials (e.g., “Reprinted [Adapted] with permission. © *2019 Traci Giuliano. All Rights Reserved*.”).

^3^In our department, the general expectation is that students will co-author publications from these capstone projects if the results are good. In research methods, publication is not an expectation, although conference presentation is fairly common, either at our campus-wide undergraduate research symposium or at a regional psychology conference; however, to motivate students on the first class day, I tell them that with good results and an excellent final paper, publication is a possibility, and I show them examples of published articles written by past students.

^4^The chapter is not included in the linked writing spiral because I do not own the copyright to this book. The publisher's website for this book can be found at https://www.pearson.com/us/higher-education/program/Scott-Psychology-Student-Writer-s-Manual-The-2nd-Edition/PGM226289.html

## Appendix

### Undergraduate^*^ Coauthored Publications

^*^Rosa, M. N., Matthews, S. J., Giuliano, T. A., ^*^Thomas, K. H., ^*^Swift, B. A., and ^*^Mills, M. M. (in press). Encouraging erotic variety: Identifying correlates of, and strategies for promoting, sexual novelty in romantic relationships. *Pers. Individ. Dif*.

^*^Butterworth, S. E., Giuliano, T. A., ^*^White, J. R., ^*^Cantu, L., and ^*^Fraser, K. C. (2019). Sender gender influences emoji interpretation. *Front. Psychol*. 10:784. doi: 10.3389/fpsyg.2019.00784

Giuliano, T. A., ^*^Johnson, S. M., ^*^Herselman, J. R., and ^*^Hutzler, K. T. (2019). “Attitudes toward polyamory scale,” in *Handbook of Sexuality-Related Measures*, 4th Edn., eds R. Milhausen, J. K. Sakaluk, T. Fisher, C. M. Davis, and W. Yarber (Thousand Oaks, CA: Sage Publications).

^*^Matthews, S. J., Giuliano, T. A., ^*^Rosa, M. N., ^*^Thomas, K. H., and ^*^Swift, B. A. (2019). “Sexual novelty scale,” in *Handbook of Sexuality-Related Measures*, 4th Edn., eds R. Milhausen, J. K. Sakaluk, T. Fisher, C. M. Davis, and W. Yarber (Thousand Oaks, CA: Sage Publications).

^*^Matthews, S. J., Giuliano, T. A., ^*^Rosa, M. N., ^*^Thomas, K. H., ^*^Swift, B. A., ^*^Ahearn, N. D., et al. (2018). The battle against bedroom boredom: development and validation of a brief measure of sexual novelty in relationships. *Can. J. Hum. Sex*. 27, 277–287. doi: 10.3138/cjhs.2017-0041

^*^Matthews, S. J., Giuliano, T. A., ^*^Thomas, K. H., ^*^Straup, M. L., and ^*^Martinez, M. A. (2018). Not cool, dude: perceptions of solicited vs. unsolicited sext messages from men and women. *Comput. Hum. Behav*. 88, 1–4. doi: 10.1016/j.chb.2018.06.14

^*^Hutzler, K. T., Giuliano, T. A, ^*^Herselman, J. R., and ^*^Johnson, S. M. (2015). Three's a crowd: Public awareness and (mis)perceptions of polyamory. *Psychol. Sex*. 7, 69–87. doi: 10.1080/19419899.2015.1004102

^*^Johnson, S. M., Giuliano, T. A, ^*^Herselman, J. R., and ^*^Hutzler, K. T. (2015). Development of a brief measure of attitudes towards polyamory. *Psychol. Sex*. 6, 325–339. doi: 10.1080/19419899.2014.1001774

^*^Blomquist, B. A., and Giuliano, T. A. (2012). “Do you love me, too?” Perceptions of Responses to ‘I love you.” *N. Am. J. Psychol*. 14, 407–18.

^*^Gomillion, S. C., and Giuliano, T. A. (2011). The influence of media role models on gay, lesbian, and bisexual identity. *J. Homosex*. 58, 330–354. doi: 10.1080/00918369.2011.546729

^*^Howell, J., ^*^ and Giuliano, T. A. (2011). The effects of expletive use and team gender on perceptions of coaching effectiveness. *J. Sport Behav*. 34, 69-81.

^*^Howell, J., ^*^Egan, P., ^*^Ackley, B., and Giuliano, T. A. (2011). The reverse double standard in perceptions of student-teacher sexual relationships: the role of gender, initiation, and power. *J. Soc. Psychol*. 151, 180–200. doi: 10.1080/00224540903510837

^*^Egan, P., and Giuliano, T. A. (2009). Unaccommodating attitudes: perceptions of students with learning disabilities as a function of accommodation use and test performance. *N. Am. J. Psychol*. 11, 487–500.

^*^Osborne, R. L, ^*^Ackley B. D, and Giuliano, T. A., (2008). The “skinny” on coffee drinkers: gender differences in healthy beverage choice. *Psi Chi J. Undergr. Res*. 13, 159–163.

^*^Riggs, A. L., & Giuliano, T. A. (2007). Running in the family or swimming in the gene pool: the role of family history and genetic risk in individuals' illness perceptions. *J. Health Psychol*. 12, 883–894. doi: 10.1177/1359105307082452

^*^Stanzer, M., Guarraci, F., Giuliano, T. A., and Sims, A. (2007). Paramedic or EMT-basic partner? study evaluates preferred partner types & the effect of partners on work-related stress levels. *J. Emerg. Med. Serv*. 32, 72–74. doi: 10.1016/S0197-2510(07)72232-5

Giuliano, T. A., ^*^Turner, K. L., ^*^Lundquist, J. C., and ^*^Knight, J. L., (2007). Gender and the selection of public athletic role models. *J. Sport Behav*. 30, 161–198.

Hebl, M. R., Giuliano, T. A., King, E. B., ^*^Knight, J. L., Shapiro, J. R., Skorinko, J. L., et al. (2004). Paying the way: the ticket to gender inequality in sports. *Sex Roles* 51, 227–235. doi: 10.1023/B:SERS.0000037766.92972.80

^*^Knight, J. L., and Giuliano, T. A. (2003). Blood, sweat, and jeers: the impact of the media's heterosexist portrayals on perceptions of male and female athletes. *J. Sport Behav*. 26, 272–284.

^*^Wilke, K. M., ^*^Turner, K. L., & Giuliano, T. A. (2003). Smoke screens: cross-cultural effectiveness of anti-smoking messages. *N. Am. J. Psychol*. 5, 431–442.

^*^Dodd E. H., Giuliano, T. A., ^*^Boutell, J. M., and ^*^Moran, B. E. (2001). Respected or rejected: perceptions of women who confront sexist remarks. *Sex Roles* 45, 567–577. doi: 10.1023/A:1014866915741

^*^Knight, J. L., and Giuliano, T. A. (2001). She's a “looker;” he's a Laker: the consequences of gender-stereotypical portrayals of male and female athletes by the print media. *Sex Roles* 45, 217–229.

^*^Knight, J. L., Giuliano, T. A., and ^*^Sanchez-Ross, M. G. (2001). Famous or infamous? The influence of celebrity status and race on perceptions of responsibility for rape. *Basic Appl. Soc. Psychol*. 23, 183–190. doi: 10.1207/S15324834BASP2303_4

^*^Dickson, A., Giuliano, T. A., ^*^Morris, J. C., and ^*^Cass, K. L. (2001). Eminem versus Charley Pride: race, stereotypes, and perceptions of rap and country music performers. *Psi Chi J. Undergr. Res*. 6, 175–179. doi: 10.24839/1089-4136.JN6.4.175

^*^Kirkendall, K. D., ^*^Dixon, D. P., Giuliano, T. A., and ^*^Raney, A. E. (2001). The bold and the beautiful: the effect of physical attractiveness and extraversion on desirability. *Psi Chi J. Undergr. Res*. 6, 180–186. doi: 10.24839/1089-4136.JN6.4.161

Giuliano, T. A., ^*^Popp, K. E., and ^*^Knight, J. L. (2000). Footballs vs. Barbies: Childhood play activities as predictors of sport participation by women. *Sex Roles* 42, 159–181. doi: 10.1023/A:1007035122089

^*^Cohorn, C. A., and Giuliano, T. A. (1999). Predictors of adjustment and institutional attachment in first-year college students. *Psi Chi J. Undergr. Res*. 4, 47–56. doi: 10.24839/1089-4136.JN4.2.47

^*^Cox, C. B., and Giuliano, T. A. (1999). Constructing obstacles vs. making excuses: examining perceivers' reactions to behavioral and self-reported self-handicapping. *J. Soc. Behav. Person*. 14, 419–432.

^*^Fiala, S. E., Giuliano, T. A., ^*^Remlinger, N. M., and ^*^Braithwaite, L. C. (1999). Lending a helping hand: the effects of sex stereotypes and gender on likelihood of helping. *J. Appli. Soc. Psychol*. 29, 2164–2176. doi: 10.1111/j.1559-1816.1999.tb02300.x

Giuliano, T. A., and ^*^Cohorn, C. A. (1997). Predictors of adjustment and institutional attachment in first-year college students. *High. Educ. Abstracts* 33, 27–28.
